# Management after acute rupture of the anterior cruciate ligament (ACL). Part 1: ACL reconstruction has a protective effect on secondary meniscus and cartilage lesions

**DOI:** 10.1007/s00167-022-06960-1

**Published:** 2022-04-20

**Authors:** Wolf Petersen, Daniel Guenther, Andreas B. Imhoff, Mirco Herbort, Thomas Stein, Christian Schoepp, Ralph Akoto, Jürgen Höher, Sven Scheffler, Amelie Stoehr, Thomas Stoffels, Martin Häner, Tilman Hees, Julian Mehl, Andree Ellermann, Matthias Krause, Natalie Mengis, Christian Eberle, Peter E. Müller, Raymond Best, Patricia M. Lutz, Andrea Achtnich

**Affiliations:** 1 Sportsclinic Berlin, Department of Orthopedics, Martin Luther Hospital, Berlin-Grunewald, Caspar-Theyß-Straße 27-31, 14193 Berlin, Germany; 2 Department of Orthopaedic Surgery, Trauma Surgery, and Sports Medicine Cologne Merheim Medical Center (Witten/Herdecke University), Ostmerheimer Str. 200, 51109 Cologne, Germany; 3 Department for Orthopedic Sports Medicine, Technical University Munich, Ismaninger Strasse 22, 81675 Munich, Germany; 4 OCM Clinic Munich, Steinerstrasse 6, 81369 Munich, Germany; 5 SPORTHOLOGICUM® Frankfurt Am Main, Siesmayerstraße 44, 60323 Frankfurt am Main, Germany; 6 Department of Sports Medicine, Goethe University Frankfurt, Ginnheimer Landstraße 39, 60487 Frankfurt am Main, Germany; 7 Department of Arthroscopic Surgery, Sports Traumatology and Sports Medicine, BG Klinikum, Duisburg gGmbH, Großenbaumer Allee 250, 47249 Duisburg, Germany; 8 Department of Trauma and Orthopaedic Surgery, Sports Traumatology, BG Hospital Hamburg, Hamburg, Germany; 9 SPORTSCLINIC COLOGNE, Ostmerheimer Str. 200, 51109 Köln, Germany; 10 Sporthopaedicum Berlin, Bismarckstrasse 45-47, 10627 Berlin, Germany; 11 OC Stadtmitte, Friedrichstraße 63, 10117 Berlin, Germany; 12 ARCUS Sports Clinic, Rastatter Str. 17-19, 75179 Pforzheim, Germany; 13 Department of Trauma and Orthopaedic Surgery, University Medical Center Hamburg-Eppendorf, Martinistraße 52, 20246 Hamburg, Germany; 14 Department of Orthopaedics and Trauma Surgery, Musculoskeletal University Center Munich (MUM), University Hospital, LMU Munich, Marchioninistraße 15, 81377 Munich, Germany; 15 Department of Orthopaedic and Sports Trauma Surgery, Sportklinik Stuttgart, Taubenheimstraße 8, 70372 Stuttgart, Germany; 16 Department of Sports Medicine and Orthopaedics, University of Tuebingen, Hoppe Seyler Strasse 5, 72074 Tuebingen, Germany

**Keywords:** Osteoarthritis, Meniscus, Cartilage, ACL, Consensus, Algorithm

## Abstract

**Purpose:**

The aim of this consensus project was to validate which endogenous and exogenous factors contribute to the development of post-traumatic osteoarthritis and to what extent ACL (anterior cruciate ligament) reconstruction can prevent secondary damage to the knee joint. Based on these findings, an algorithm for the management after ACL rupture should be established.

**Methods:**

The consensus project was initiated by the Ligament Injuries Committee of the German Knee Society (Deutsche Kniegesellschaft, DKG). A modified Delphi process was used to answer scientific questions. This process was based on key topic complexes previously formed during an initial face-to-face meeting of the steering group with the expert group. For each key topic, a comprehensive review of available literature was performed by the steering group. The results of the literature review were sent to the rating group with the option to give anonymous comments until a final consensus voting was performed. Consensus was defined a-priori as eighty percent agreement.

**Results:**

Of the 17 final statements, 15 achieved consensus, and 2 have not reached consensus. Results of the consensus were summarized in an algorithm for the management after ACL rupture (infographic/Fig. 2).

**Conclusion:**

This consensus process has shown that the development of post-traumatic osteoarthritis is a complex multifactorial process. Exogenous (primary and secondary meniscus lesions) and endogenous factors (varus deformity) play a decisive role. Due to the complex interplay of these factors, an ACL reconstruction cannot always halt post-traumatic osteoarthritis of the knee. However, there is evidence that ACL reconstruction can prevent secondary joint damage such as meniscus lesions and that the success of meniscus repair is higher with simultaneous ACL reconstruction. Therefore, we recommend ACL reconstruction in case of a combined injury of the ACL and a meniscus lesion which is suitable for repair.

**Level of evidence:**

Level V.

## Introduction

The ACL is the primary stabilizer against anterior tibial translation and internal tibial rotation [26]. Therefore, in the case of an ACL rupture, anterior and rotational instability can occur, which leads to functional impairment of the patient.

If chronic instability develops after an ACL rupture, the changed kinematics result in an increased mechanical load on the menisci and cartilage with the risk of secondary damage. To restore the impaired kinematics of the knee, the torn ACL can be replaced surgically with an allogenic or autologous tendon graft [25].

However, the statements in the literature regarding the best management after ACL rupture are contradicting. Some authors favour surgical treatment while others favour a non-surgical approach [7, 12, 14, 21, 30]. An important factor that plays a role in the indication for ACL reconstruction (ACL-R) is to what extent this operation slows down further secondary damage and the progression of post-traumatic osteoarthritis (OA) of the knee. Although the primary goal of ACL-R is to regain stability, many doctors see the protective effect of an operation on the development of OA as an important indication criterion.

So far, the protective value of an ACL-R has been viewed as controversial, as various studies have shown that post-traumatic OA of the knee can develop despite an operation. For example, Poulsen et al. and Luc et al. found in a meta-analysis that the percentage of patients with knee post-traumatic knee OA was even increased in patients who underwent ACL-R in comparison to patients with ACL deficiency [17, 28]. From these data, it was concluded that the current literature does not provide any evidence to suggest that ACL-R is an adequate intervention to prevent post-traumatic OA of the knee [17].

However, a question as complex as the development of OA after ACL injury cannot be answered by a single study, systematic review, or meta-analysis, as these studies mostly focus on one outcome criterion. The development of post-traumatic OA of the knee after injury to the ACL depends on several internal and external risk factors. Scientifically based consensus processes are more suitable to detect complex correlations, discuss and adopt recommendations, and answer clinically relevant questions [7, 8].

The first algorithm from this consensus project of the Committee for Ligament Injuries of the German Knee Society (DKG) aims to validate which endogenous and exogenous factors contribute to the development of post-traumatic OA and to what extent ACL-R can prevent secondary damage to the knee joint. Aim of this project was to create a consensus based scientific infographic to demonstrate the relationships between ACL injuries and secondary damage.

## Materials and methods

**Fig. 1:**
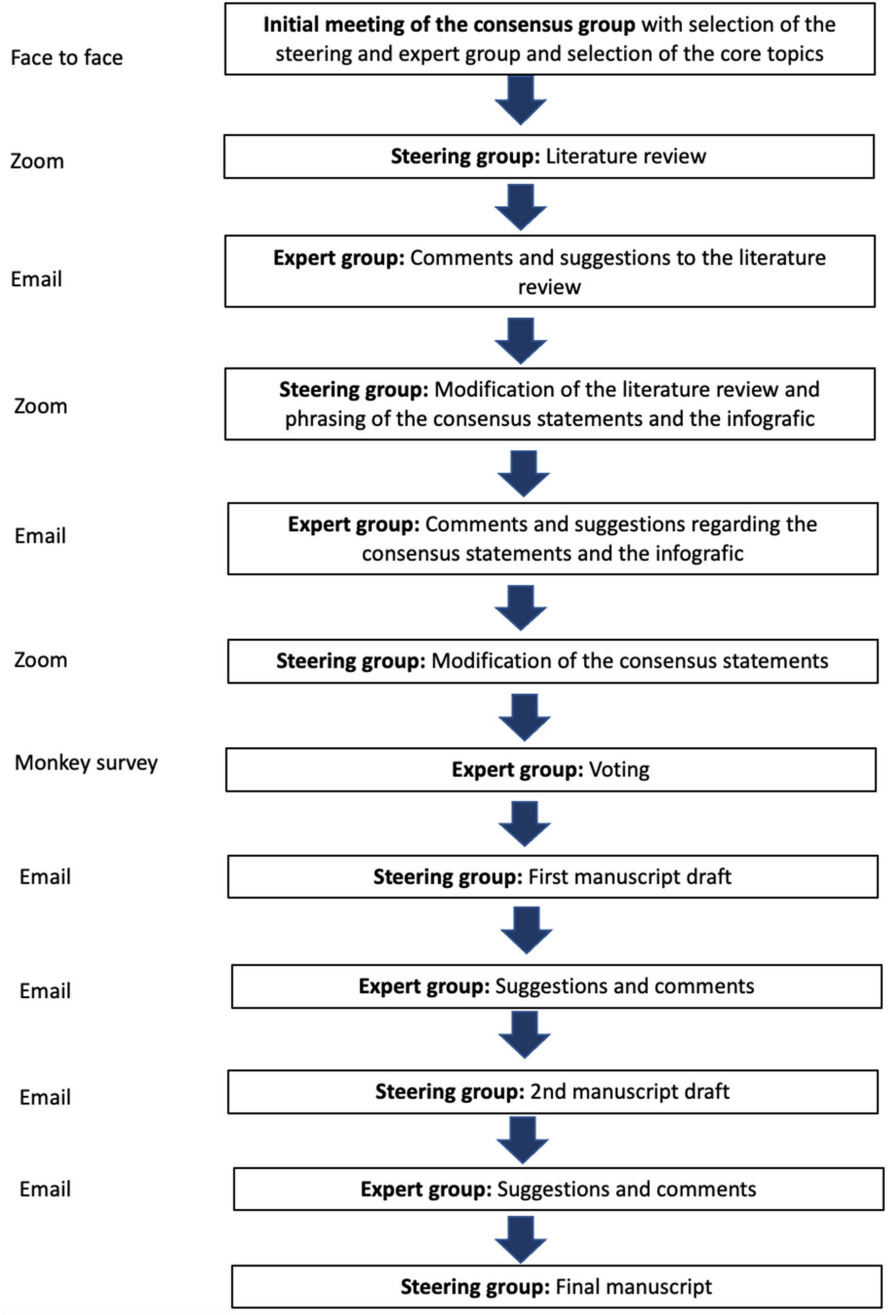
This schematic drawing shows the procedure during the consensus process

At the beginning of this modified Delphi consensus process, a steering and a rating group were formed (Fig. 1). The steering group was elected during an initial face-to-face meeting by all members of the project. Both groups were recruited from the ligament committees of the German Knee Society (DKG) and the Society of Joint Surgery and Arthroscopy (AGA). All members of the steering and rating group were experts in knee surgery (certified knee surgeon of the German Knee Society) with scientific experience (minimum of two published scientific articles). The steering group consisted of three people, and the rating group consisted of 21 people.

In the initial face-to-face meeting, the steering group together with the rating group formed various key topic complexes for which various questions were formulated.

For each key topic, a comprehensive review of the literature using the following scientific data bases (Pubmed, Google Scholar, Scopus, and Cochrane) was performed by the steering group to answer the questions. For each topic, specific search terms were defined. Meta-analysis, systematic reviews, and clinical studies with evidence levels between I and V were included in this analysis. The period 2010–2020 was selected for the literature search. This period was chosen because this period represents the current literature relevant to answering the research questions. If a meta-analysis or a systematic review had already been published, a separate analysis of individual studies was performed.

The results of the literature review were briefly summarized for each question by the steering group, and this summary was made available to the expert group in a first round with the request for comments and additions. The answers of the expert group were sent anonymously by email. Based on these comments and remarks, the literature reviews were then supplemented and modified by the steering group. On the basis of the modified reviews, answers to the questions were then formulated and presented to the expert group in a further round.

Comments and suggestions were again sent individually to the steering group by each member of the Expert Group. A further modification of the consensus statements was then made on the basis of the suggestions. This procedure was repeated several times for each question. Whenever a response was not received for more than two weeks, reminder emails were sent. Sufficient consensus was defined a-priori as eighty percent agreement. Statements with less than 80% approval were included in the consensus paper, noting the corresponding approval value [8].

The present article describes the first topic complex with the aim to clarify the role of ACL injury on the development of secondary damage. Ten key questions should be answered (see headings in the result section):

The definition of chronic instability for the present consensus process included the time interval between an ACL injury and the trauma (> 3 months) or the occurrence of more than episode of giving way after the ACL injury. Post-traumatic OA was defined as degenerative joint disease following an injury to the knee. The primary meniscus lesion was defined as a meniscus lesion that occurred directly on the first trauma in which the ACL injury was also caused. A meniscus lesion that occurred in the period after the initial trauma was defined as a secondary meniscus lesion. For statement 1a and 1b, the term “often” was defined as a frequency of more than 50%.

The respective scientific evidence was given at the end of each statement. The questionnaires were created using the SoSci program, SoSci Survey GmbH, Munich, Germany. The questionnaires were sent by mail, and the data was processed anonymously. The completeness of the questionnaires could be traced automatically. The data was processed using SPSS (Version 20.0; IBM) and Excel 2019 (Microsoft Corporation).

After the last vote, the steering group developed an infographic that summarized the results. The infographic was then sent to the expert group with a request for suggestions for changes and comments (Fig. 1). Due to the various comments, a first modification of the infographic was made by the steering group. The modified infographic was then sent to the steering group with a new request for suggestions for changes and comments. A final version of the infographic was then created on the basis of these comments and sent to the expert group for final voting.

## Results

**Fig. 2:**
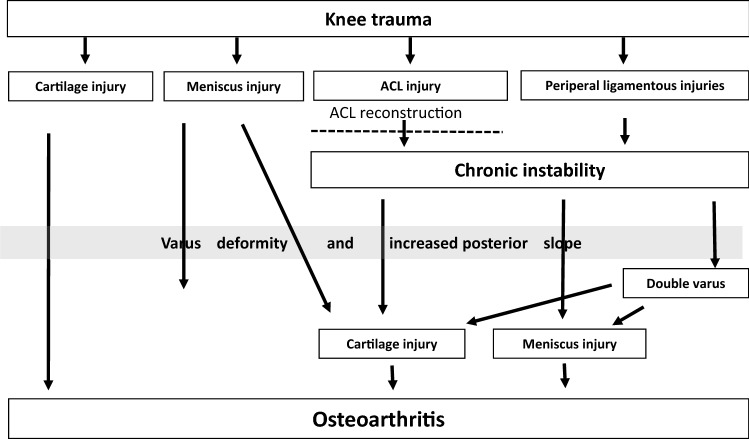
Scientific infographic summarizing the results of the consensus process. Agreement: 100%; ACL: anterior cruciate ligament; double varus: Combination of ligamentous varus due to lateral laxity and osseous varus due to decreased mechanical medial tibial angle or increased mechanical distal femoral angle

Of the 17 final statements, 15 achieved consensus and two did not achieve consensus. The infographic (Fig. 2) summarizes the results of the consensus process.

1. Does knee trauma with ACL rupture result in primary meniscus and cartilage damage?

A systematic review analysed various studies in which meniscus and cartilage damage in early ACL-R (primary damage) was compared with that after delayed ACL-R [20]. Thirty eight of the 40 studies included in this systematic review showed that in the context of an acute ACL rupture, accompanying meniscal injuries can occur [20]. The rate of primary meniscus injury varied between 35 and 100%. Most of these studies showed that the lateral meniscus was affected more often than the medial in acute ACL injuries [20].

Cartilage damage is a frequent primary accompanying lesion of the acute ACL lesion [20]. For ACL-R within the first six months after trauma, the mean incidence of cartilage damage was 33% [20].

In a prospective MRI study in which the MRI was performed immediately after trauma, a cartilage lesion was detectable in all cases after an acute ACL lesion [27]. However, this study includes occult damage that is arthroscopically undetectable.

Consensus statement 1a: In the case of knee trauma with ACL injury, there is often accompanying primary meniscus damage (Level of evidence: IV).

Agreement: 95%

Consensus statement 1b: In the case of knee trauma with ACL injury, there is often accompanying primary cartilage damage (Level of evidence: IV).

Agreement: 73%

2. Does chronic instability after ACL rupture lead to secondary meniscus and cartilage damage?

In a recent systematic review, 35 out of 40 studies (88%) showed evidence of a positive correlation between the rate of meniscus and/or cartilage lesions and the time since ACL injury [20]. This correlation was more evident for the medial meniscus than the lateral meniscus [20]. In particular, a delay of more than 6 months was critical for secondary medial meniscus injuries [risk ratio 0.58 (95% CI 0.44–0.79)] and a delay of more than 12 months was critical for cartilage injuries [risk ratio 0.42 (95%) CI 0.29–0.59)] [20].

Another systematic review by Sommerfeld et al. has shown that chronic instability after ACL rupture favors the development of degenerative changes in the knee joint [31]. Six of seven studies reported a positive association between recurring instability events and medial meniscus damage [OR range 3.46 (95% CI 1.24–9.99) to 11.56 (1.37–521.06)] [31]. The relationship between instability episodes and lateral meniscus or cartilage damage, however, was inconsistent [31].

Consensus statement 2a: Chronic instability following ACL lesion can lead to secondary medial meniscal lesions (Level of evidence: IV).

Agreement: 100%

Consensus statement 2b: Secondary lateral meniscus lesions also occur after ACL lesion (Level of evidence: IV).

Agreement: 100%

Consensus statement 2c: Chronic instability is a cause of secondary lateral meniscus lesions (Level of evidence: IV).

Agreement: 100%

Consensus statement 2d: Secondary cartilage damage occurs in the course of ACL injury (Level of evidence: IV).

Agreement: 95%

Consensus statement 2e: Chronic instability is likely to be the cause of secondary cartilage damage (Level of evidence: IV).

Agreement: 100%

Consensus statement 2.f: Other factors, independent of the instability, as the cause of secondary cartilage damage after ACL lesion are possible (Level of evidence: IV).

Agreement: 95%

3. Is an ACL lesion a risk factor for post-traumatic OA of the knee?

Various systematic reviews have shown that the rupture of the ACL is a risk factor for the development of post-traumatic OA of the knee [1, 5, 15, 17, 28, 32]. In these reviews, OA rates after ACL injury varied between 13.0% and 79.6%. Patients treated both surgically and non-surgically were included in these systematic reviews.

Most studies were included in a recent systematic review by Poulsen et al. [28]. In this systematic review, 53 studies with approximately one million participants were included (185 219 participants with ACL injury) [28]. The odds of developing knee OA following ACL injury are approximately four times higher compared to a non-injured knee [28]. After fifteen years, the cumulative incidence of knee arthroplasty following cruciate ligament reconstruction was low (1.4%). However, it was seven times greater than the cumulative incidence of knee arthroplasty among matched control patients from the general population (0.2%) [28].

Consensus statement: Sustaining an ACL lesion is a risk factor for the development of post-traumatic OA of the knee. (Level of evidence: IV).

Agreement: 100%

4. Are primary and secondary meniscus lesions a risk factor for post-traumatic OA of the knee after ACL injury?

Various systematic reviews were able to identify the accompanying meniscus lesion as an important risk factor for the development of post-traumatic OA of the knee [5, 15, 19, 28].

In a systematic review by Claes et al., patients with ACL-R and menisectomy had a prevalence of higher-grade signs of OA (IKDC C and D) of 50%. In patients without a menisectomy, the prevalence of higher-grade signs of OA was only 16% [5].

Van Meer et al. could only show an association between medial meniscus damage and post-traumatic OA; for lateral meniscal damage, this association could not be demonstrated in this systematic review [19]. Luc et al. were able to show that patients with an accompanying menisectomy in particular benefited from an ACL-R [17].

Consensus statement: Primary and secondary meniscus lesions increase the risk of post-traumatic OA (Level of evidence: IV).

Agreement: 100%

5. Is primary and secondary cartilage damage a risk factor for post-traumatic OA of the knee after ACL injury?

In their systematic review, Van Meer et al. were able to identify eight studies that examined the influence of cartilage damage on post-traumatic OA of the knee [19]. According to this information, there is limited evidence for an influence of medial and femoropatellar cartilage damage on the development of post-traumatic OA of the knee after an ACL lesion [19]. In this review, there was limited evidence that lateral cartilage damage was not a risk factor for post-traumatic OA of the knee after an ACL lesion [19].

A registry study also showed that the Knee Injury and Osteoarthritis Outcome Score (KOOS) after ACL-R in patients with full-layer cartilage damage were worse than in patients without pre-existing cartilage damage [34].

Consensus statement: Primary and secondary cartilage damage increase the risk of post-traumatic OA (Level of evidence: IV).

Agreement: 100%

6. Is varus deformity a risk factor for post-traumatic OA of the knee after an ACL lesion?

Only one prospective observational study is available on the importance of the leg axis for the development of post-traumatic OA of the knee after an ACL lesion [33]. In this study, 100 patients were followed up for a 15-year period after a complete ACL lesion. To determine the influence of the osseous varus deformity on the post-traumatic OA of the knee after an ACL lesion, the post-traumatic OA of the knee was set in relation to the leg axis of the uninjured opposite side. The results showed that varus alignment of the uninjured knee joint was associated with development of OA in the injured knee joint after 15 years [33].

Consensus statement: Varus deformity is a risk factor for post-traumatic OA after ACL lesion (Level of evidence: IV).

Agreement: 95%

7. Can secondary meniscus and cartilage lesions be prevented by ACL reconstruction?

Two systematic reviews showed a protective effect of ACL-R on secondary meniscal lesions [4, 13].

Chalmers et al. included 40 cohort studies with a mean follow-up of 13.9 years in their systematic review [4]. Secondary meniscus operations were significantly less common in patients whose ACL tears were surgically treated when compared to ACL tears treated non-operatively (13.9% vs. 29.4%) [4],

Korpershoek et al. were able to identify one randomized controlled and 27 level 3 and 4 studies in which a protective effect of an ACL-R on the development of secondary meniscus lesions could be shown [13]. In this review, there was evidence (level 3) that early ACL-R (within three months after injury) protects the meniscus [13].

No studies are available on the protective effect of ACL-R on the development of secondary local cartilage damage.

Consensus statement 7a: Secondary meniscus lesions can be prevented by ACL-R (Level of evidence: IV).

Agreement: 100%

Consensus statement 7b: No studies are available on the protective effect of ACL re-construction on the development of secondary local cartilage damage (Level of evidence: IV).

Agreement: 73%

8. Are meniscus lesions more suitable for repair with early ACL-R than with later surgical treatment?

A recent systematic review showed that the prognosis of a meniscus suture is better with early ACL-R than with later surgical treatment [15].

In a further systematic review, seven studies were able to show that the rate of meniscal suture repair decreased significantly with the time to ACL rupture [20]. The most common cutoff values that correlated with a poorer chance of meniscus repair were six weeks (*n* = 3 studies), followed by two months (*n* = 2 studies), three weeks (*n* = 1 study) and six months (*n* = 1 study). Later on, the rate of meniscus reparations decreased significantly.

Another more recent systematic review confirmed these findings [13]. Meniscus repair failure was observed less often in patients with ACL-R than in patients with ACL deficiency [13].

Consensus statement: The rate of meniscus refixation is higher with early ACL-R than with later ACL -R (Level of evidence: IV).

Agreement: 100%

9. Does ACL-R protect the repaired meniscus?

A systematic review examined the extent to which an ACL-R protects a repaired meniscus [13]. The success rate of meniscus repair was found to be better in eight level 3 and 4 studies when the ACL-R was performed at the same time as the meniscus repair. For example, in one study, only 14.5% meniscus repairs failed when performed together with ACL reconstruction, whereas 27% of meniscus repairs failed when performed without ACL-R (*p* < 0.05) [13].

Consensus statement: The success of meniscus repair is higher with simultaneous ACL-R than without (Level of evidence: IV).

Agreement: 95%

10. Can post-traumatic OA of the knee be prevented with an ACL reconstruction?

In most of the systematic reviews, due to the heterogeneity of the included studies, no difference could be found between surgically and non-surgically treated patients (different follow-up, different radiological OA scores, different surgical techniques, etc.) [9, 39].

In their systematic review, Luc et al., however, found a protective effect with regard to OA only for patients with an accompanying meniscectomy [17].

Ajuied et al. included nine studies in a systematic review, which had a 10-year follow-up and analysed knee OA with the Kellgren and Lawrence score [1]. In meta-analysis, of 6 studies the OA rate of surgically and non-surgically treated patients 10 years after ACL lesion was only 20.3%, compared to an OA rate of 4.9% for uninjured patients. After an ACL reconstruction, the relative risk for OA was only 3.62 in contrast to 4.98 for patients with non-surgical therapy. However, studies with non-anatomical reconstruction techniques were included in this review [1].

Rothrauf et al. carried out a systematic review on the question of OA rates after ACL-R and divided the reconstruction techniques into anatomical and non-anatomical reconstruction techniques [29]. On a normalized OA classification scale, 23.2% of the patients after anatomical ACL-R and 43.9% of the patients after non-anatomical ACL-R had knee OA [29].

Consensus statement: There is evidence that an anatomical ACL-R can reduce the risk of developing post-traumatic OA of the knee after an ACL lesion (Level of evidence: IV).

Agreement: 95%

## Discussion

There was 100% agreement that primary and secondary damage of the menisci and/or cartilage increase the risk of post-traumatic OA after ACL injury. The relationship between accompanying meniscus damage and the development of OA of the knee after ACL injury has been well investigated [5]. Another systematic review was able to confirm the positive effect of meniscus repairs on the OA process [23]. It can, therefore, be concluded that meniscal injuries in combination with ACL rupture should be repaired, if possible, to reduce this risk factor for OA. If there is an indication for meniscus repair, an ACL-R should be considered as well because meniscus repair failure occurs less often in patients with ACL-R than in patients with ACL deficiency [13]. There was a strong consensus that meniscus injuries are more suitable for repair in the setting of an early ACL-R than in later surgical treatment and that ACL-R protects meniscal repair because there is evidence that the prognosis of a meniscus repair is better with early ACL-R than with later surgical treatment [15].

With regard to the surgical treatment of meniscus injuries, it also appears to be relevant that the menisci are important secondary stabilizers in the anterior tibial translation and thus support the ACL [16]. Lateral meniscus root tears and the medial ramp lesions should be emphasized and strongly considered for operative treatment, as both types of tear pattern significantly impair the stability of the knee joint [11, 19].

In contrast to the meniscus, only limited data is available on the influence of primary cartilage lesions on the development of post-traumatic knee OA [1]. This fact explains why two consensus statements regarding the role of cartilage damage in the ACL injured knee failed to reach a consensus of 80%. For statement 1b (high frequency of cartilage damage after ACL injury) the steering group reached a consensus of only 73%. This voting result can be explained with the discrepant findings in the literature. In a systematic review, the incidence of cartilage damage six months after an ACL injury was 33%, whereas in another study, occult cartilage damage detectable by MRI was 100%. The statement “no studies are available on the protective effect of ACL-R on the development of secondary local cartilage damage” also received only 73% agreement. This disagreement could be explained by the fact that the discrimination between cartilage damage and OA is often blurred. Further studies are necessary here, in particular to examine the influence of surgical procedures for cartilage repair on the risk of OA.

Primary damage accompanying ACL injury is relevant for the development of post-traumatic OA of the knee, and secondary injuries to the menisci and cartilage also play an important role. The fact that secondary damage increases in quantity and severity with the time interval to the knee trauma leads to the assumption that this is likely caused by instability-related disrupted kinematics [20]. In secondary meniscus injuries, the posterior horn of the medial meniscus is usually affected, which is more stressed by the increased anterior tibial translation [24]. A biomechanical study has shown that in the ACL deficient knee, the posterior horn of the medial meniscus encounters shear forces due to increased translation of the menisco-tibial complex in relation to the femoral condyle [9].

In light of these findings, it seems relevant that there is evidence that secondary meniscal and cartilage injuries can be prevented by ACL-R [4, 30]. There is also a strong consensus for this causal relationship in the present study.

With regard to posttraumatic OA, previous papers have paid little attention to accompanying osseous deformities. To our knowledge, there is only one prospective observational study available on the importance of varus deformity for the development of unicompartmental OA after an ACL lesion [33]. However, varus deformity appears to be an important risk factor as the prevalence of varus deformity in the general population is high. A cross-sectional study in 250 healthy volunteers has shown that 32% of men and 17% of women have constitutional varus knees with a mechanical alignment of 3° varus or more [2]. The importance of varus deformity is that it is a known risk factor for a medial meniscus lesion [10]. Therefore, in patients with chronic ACL injury and varus deformity, there are two factors that place increased stress on the medial meniscus. Considering the key role that the meniscus lesion plays in the development of post-traumatic OA of the knee, there was strong consensus in the present study that varus deformity is a disease-modifying factor for the patient with chronic ACL injury. A vicious circle can arise here because it has been shown that varus alignment aggravates tibiofemoral contact pressure rise after sequential medial meniscus resection [35].

All of these factors must be considered when discussing the importance of ACL-R for the prevention of post-traumatic OA of the knee. It is therefore not surprising that so far, there is low-level evidence that ACL-R can stop the progression of OA after ACL injury. In most of the previous systematic reviews no difference could be found between surgically and non-surgically treated patients [19, 39]. However, it should not be concluded that ACL-R has no effect on the progression of OA. The present consensus project has shown that the development of post-traumatic OA of the knee is a multifactorial process. Accompanying injuries, secondary damage, and endogenous factors (bony deformities) play an important role. Another factor that complicates the interpretation of previous studies on ACL-R and OA is the relevant heterogeneity in previous studies (different surgical techniques, follow up, and scores). More high-quality research is certainly necessary. Nevertheless, there is consensus in the present study that an anatomical ACL-R helps to slow down the progression of post-traumatic OA, as there is evidence that ACL-R prevents secondary damage to the meniscus and cartilage [4]. Recent studies have also shown that the effect on OA progression was better with anatomical reconstruction techniques than with non-anatomical reconstruction techniques [29].

The results of this consensus process have clinical relevance. Preservation of the meniscus should be a major goal in the treatment of ACL injuries, and the results of the present consensus process suggest that ACL-R should be considered if there is a combined injury of the anterior cruciate ligament and the medial or lateral meniscus. This is especially true if the meniscus appears to be repairable, as the failure rate of a meniscus suture is significantly lower when it is performed in combination with an ACL reconstruction. Therefore, in case of a combined ACL injury and repairable meniscus tear early ACL-R is recommended. With regard to the indication, it is also relevant that an ACL-R is suitable to prevent secondary meniscus, cartilage damage. A protective effect for the joint can, therefore, be ascribed to this operation. This effect is particularly relevant for younger patients.

As all scientific formats, a consensus process has some limitations [6]. The fact that there is a scientific consensus is no guarantee for the truth of the state of the art in science. For each scientific consensus, social and personal motives can play a role. When interviewing a group, a specific social situation arises. This format can lead to distortions due to authority or personal trench warfare, which should ideally be prevented by the anonymous e-mail survey [6]. Nevertheless, a certain amount of interaction and communication within the expert group cannot always be prevented, and the initial selection of the core topics for the consensus process took place during a face-to-face meeting of the entire group. The expert group consisted exclusively of orthopedic surgeons. Theoretically, there could therefore be a tendency to overestimate the value of operative care. Another limitation of the present study is that all of the statements on which consensus was reached was level IV evidence. However, too much attention is given to randomized controlled trials in the evidence hierarchy because not all research questions can be answered by RCTs.The weaknesses of RCTs are, for example, that rare effects and long-term effects are difficult to detect.

## Conclusion

In conclusion, this consensus statement shows that endogenous (primary and secondary meniscus lesions) and exogenous factors (varus deformity) play a decisive role for the development of post-traumatic OA of the knee after ACL injury. Therefore, ACL-R cannot always halt post-traumatic OA of the knee. However, there is evidence that ACL-R can prevent secondary joint damage such as meniscus lesions. These relationships are important factors with regard to the indication and timing of surgical treatment after an ACL injury. By developing a scientifically based and consensus-supported algorithm, these complex relationships should be made easier to understand.
